# Nesting and foraging behavior of *Xylocopa valga* in the Ejina Oasis, China

**DOI:** 10.1371/journal.pone.0235769

**Published:** 2020-07-09

**Authors:** Chunling He, Chaodong Zhu

**Affiliations:** 1 Forestry College, Henan University of Science & Technology, Luoyang, China; 2 Key Laboratory of Zoological Systematics and Evolution, Institute of Zoology, Chinese Academy of Sciences, Beijing, China; Ben-Gurion University of the Negev, ISRAEL

## Abstract

*Xylocopa valga* is extinct in Latvia and Lithuania and is critically endangered in Poland, and its distribution in the Ejina Oasis, China, is currently unknown. Studies on the biology of *X*. *valga* are scarce, and thus, conservation efforts for this species are currently limited. Here, we investigated the morphological characteristics, nest architecture, nest structure and food type of offspring in the nest cells of *X*. *valga*. This research was conducted in the *Populus euphratica* forest reserve in the Ejina Oasis, China, between July 2014 and June 2019. The primary investigation methods included visual inspection, photography, observation and measurements of nest anatomy, and examination of pollen stores by microscopy. We found that in the Ejina Oasis, China, *X*. *valga* builds its nests in the dead wood of *P*. *euphratica*. *X*. *valga* is univoltine. Its lifestyle varies from solitude to symbiosis. When many females nest near each other, several females may share a single nest entrance, based on which they build their own cells. The nests are branched. According to our results, there is a significant difference between the thickness of the inner cell partition and that of the outermost cell partition in the branched tunnel. In the *P*. *euphratica* forest area, the food for the progeny of *X*. *valga* is mainly composed of the pollen and nectar of *Sophora alopecuroide* and *Populus euphratica*. Therefore, *X*. *valga* and *S*. *alopecuroides* exhibit close ecological interactions in the *P*. *euphratica* forest ecosystem.

## Introduction

*Xylocopa*, the members of which are known as large carpenter bees, is an important genus in the Apidae family [1–6]. In recent years, *Xylocopa* populations have been reduced or even become critically endangered and gone extinct because of habitat resource reductions and damage [7–9]. Insight into the nesting habits and foraging activities of these species is essential for habitat recovery.

There are 31 *Xylocopa* subgenera distributed worldwide, which include 470 species [1, 10]. Numerous studies on the nesting biology of carpenter bees in the genus *Xylocopa* have been reported. The time of nesting and the selection of the nesting substrate are related to the species and distribution of carpenter bees [11]. For example, *Xylocopa ordinaria* [11], *X*. *pubescens* [12, 13] and *X*. *cearensis* [14] may nest in a variety of plant materials, exhibiting a wide range of nesting substrates. In contrast, *X*. *virginica*, *X*. *ciliata* and *X*. *artifex* show some specificity in their selection of nesting substrate [15]. *X*. *abbreviata* nests only on *Encholirium spectabile* of the family *Bromeliaceae* [16]. Therefore, the availability of dead wood with a suitable density can determine the distribution and abundance of *Xylocopa* species [1, 15]. Most nests of carpenter bees exhibit a clustered distribution [15, 17], which is related to the homing behavior of carpenter bee species [18] or nesting substrate resources [11, 12, 15].

The nest serves as a shelter as well as a food storage location for bees, and it is a necessary structure for bees to raise their offspring [1, 19]. With the exception of *Proxylocopa* species, which nest in the soil, *Xylocopa* species nest in dead wood, hollow stalks and bamboo [3, 7, 20–22]. The preferred nesting location and substrate have an impact on the distribution and genetic structure of *Xylocopa* populations [11, 23, 24]. In addition, the diameter of the nesting substrate and nesting substrate resources define the nest structure of carpenter bees [25, 26]. *Xylocopa* bees build nests in botanical materials, and their nests are classified into two structural types: the linear unbranched (straight chain) type and the linear branched type [1, 19]. Linear unbranched cells are cells continuously distributed along the main tunnel. Generally, the cells extend in one direction or two directions from the nest entrance. When the position of the cell being built in one direction is restricted, carpenter bees will continue to extend the building of cells in the other direction. The carpenter bees that build their nests in hollow stalks and bamboo belong to this category [15, 21, 27]. In the linear branched type of nest construction, there are branches in the main tunnel, and cells are distributed continuously along the branched tunnels. The nests that most carpenter bees build in dead wood belong to this type [22, 25, 27].

**Fig 1:**
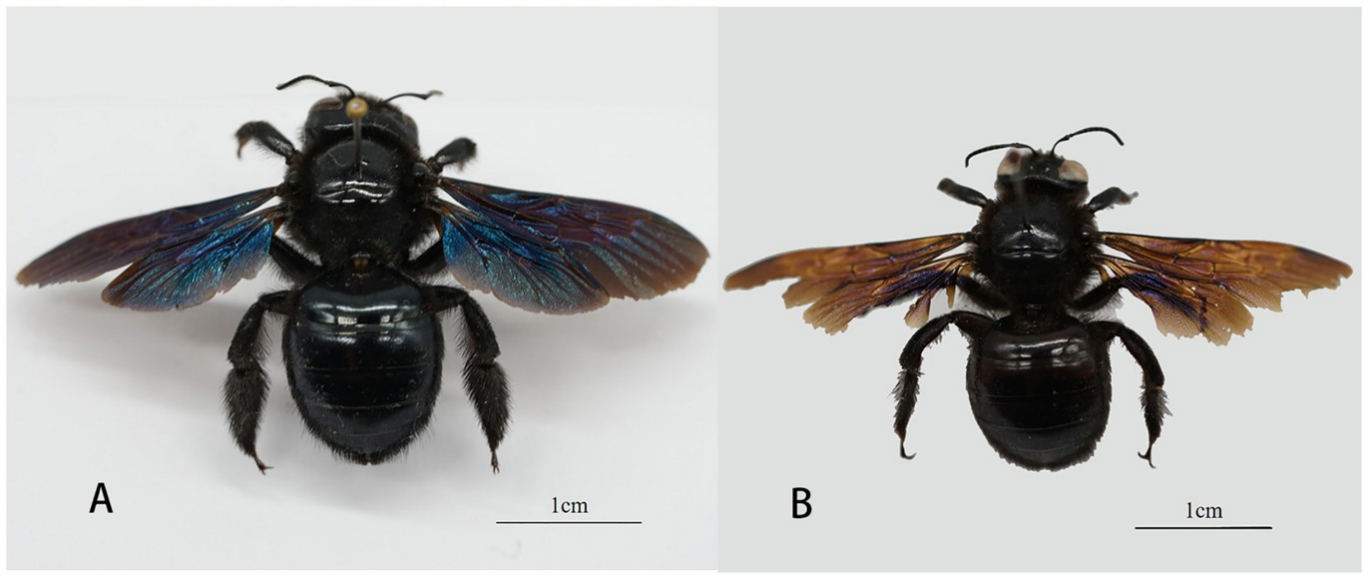
The morphology of the large carpenter bee *X*. *valga*. A. Female; B. Male.

*Xylocopa valga* is one of eight species in the *Xylocopa* subgenus of the *Xylocopa* genus [10, 19]. Males and females of *X*. *valga* are similar in morphology (Fig 1). Females are black, and their wings display a purple luster. The mandible contains 2 teeth. The two spines on the outer apex of the hind tibia account for 2/3 of the tibial length, and verrucous protrusions are found on the hind tibia [19]. The male body is also black, and the wings are brownish-purple in color. There is no verrucous protrusion on the hind tibia of males. With the exception of a small amount of black hair on the anterior surface of the head, the body is covered with brownish-black hair [19]. *X*. *valga* is distributed in northern Europe, North Africa and Asia. In China, *X*. *valga* is the only species in the *Xylocopa* subgenus [19] and is mainly found in Xinjiang, Inner Mongolia, Gansu, Tibet and other northwestern regions [10, 19]. According to the International Union for Conservation of Nature (IUCN), *X*. *valga* is extinct in Latvia and Lithuania, critically endangered in Poland, endangered in Germany and Slovenia, and vulnerable in Moldova, Switzerland and Ukraine [7]. Previously, *X*. *valga* was recorded as building its nests in dead wood or wood structures [8, 19], and nesting locations of the species can be found in standing dead wood, such as wooden electric poles and indoor wooden beams [8]. When there is a lack of dead wood in its habitat, *X*. *valga* may choose to nest in thick soil with a southern exposure [8]. *X*. *valga* has been reported to forage on 95 plant species in 30 families [8]. Nevertheless, compared with those on other *Xylocopa* species, reports on *X*. *valga* are rare. In addition, to the best of our knowledge, no studies have reported on the nesting and foraging behaviors of *X*. *valga* distributed in China.

In this study, the nesting and foraging behaviors of *X*. *valga* in the *Populus euphratica* forest area in the Ejina Oasis, China, were analyzed by observing and dissecting the cells of *X*. *valga* and observing its food and pollen under a microscope. The results of this study lay a foundation for studying the role of *X*. *valga* as a pollinator and protecting this species in the *P*. *euphratica* forest ecosystem.

## Methods

### Research site

This research was conducted in the *P*. *euphratica* forest reserve of the Ejina Oasis, Inner Mongolia, China. Ejina Banner is under the jurisdiction of the Alxa League of the Inner Mongolia Autonomous Region in the center of the inland arid region of Asia, exhibits a strong continental climate and is located in an arid desert area. The geographical coordinates are 97°10′23″-103°7′15″E, 39°52′20″-42°47′20″N, and its total land area is 114,600 km^2^. Only 23,000 hm^2^ of *P*. *euphratica* forest currently remains. The annual average temperature is 5.7–9.3 °C, and the annual average precipitation is 38.2 mm [28, 29].

In the *P*. *euphratica* forest reserve, the landscape is generally composed of desert and oases; *P*. *euphratica* is the dominant vegetation in the oases, where it grows sparsely. Other vegetation includes *Sophara alopecuroides*, *Tamarix ramosissima*, *Nitraria sibirica*, *Achnatherum splendens*, *Sphaerophysa salsula*, *Peganum harmala*, *Lycium ruthenicum*, *Alhagi sparsifolia*, *Kalidium foliatum*, *Salsola collina*, *Taraxacum mongolicum*, *Phragmites australis*, *Suaeda lossinskyi* and *Ephedra przewalskii*. Among these species, *S*. *alopecuroides* is a pioneer species used for wind erosion prevention and sand fixation within and at the edge of the *P*. *euphratica* forest [28, 30].

The field sampling and measurements conducted in this study were approved by the Environmental Protection Bureau of Ejina Banner (no. E20140305A).

### Measurement of morphological characteristics

We captured homebound *X*. *valga* bees in the vicinity of their cells in the *P*. *euphratica* forest reserve in the Ejina Oasis, Inner Mongolia, from June 1 to June 10, 2019. The morphological characteristics of the bees, including their wingspan, body length, mesothorax width, head length, head width, and proboscis length, were measured using a Vernier caliper with an accuracy of 0.01 mm.

### Observation of nesting locations and cell structure

From July 1 to July 20, 2014; July 21 to July 31, 2015; and May 22 to June 10, 2019, we carried out field exploration in the *P*. *euphratica* forest reserve and found *X*. *valga* bees flying around the dead wood of *P*. *euphratica*. We left marks at the sites of dead wood and then randomly selected dead wood lying on the ground to observe and record the nesting locations of *X*. *valga* within the dead wood and their nest entrances through visual inspection, photography, and video recordings.

We dissected 20, 3, and 20 nests in 2014, 2015 and 2019, respectively. Dissection was performed as follows. The surface bark was carefully cut open with a knife, and chips and dust were removed. A Vernier caliper was used to measure the length and width of the nest entrance [22]. The digging direction inside the nest was observed, and the corresponding tunnel was opened carefully. Once the nests were open, the following parameters were measured with a Vernier caliper: the total length of the tunnel, the number of branches in the tunnel, the length and cell number of each branched tunnel, the length and width of each cell, and the thickness of the partitions between cells. We simultaneously recorded the developmental stage of *X*. *valga* (from eggs to larvae, pupae and adults) within the cells [23] as well as the degree of wear on adult wings to determine the life history of *X*. *valga* [22, 23, 31].

### Identification of pollen species in bee food

Between June 1 and June 10, 2019, flowering plants within the foraging zone of *X*. *valga* in the *P*. *euphratica* forest in the Ejina Oasis were collected (9 plants in total). Buds that were about to bloom were collected and placed in a clean sulfuric acid paper bag. Then, the specimens were brought back to the laboratory and placed in an incubator at 25 °C for 6 h. Pollen was picked up with a dissecting needle and transferred to a 1.5 ml centrifuge tube. Acetone solution was added, followed by shaking for even mixing. The pollen solution was then dropped onto a glass slide with a pipette. The morphology of the pollen of different plants was observed with an Olympus STM6 optical microscope (×40), and these morphological data were used as a reference for the identification of pollen on the basis of morphology in the food of *X*. *valga* bees [32, 33].

During the same period, 18 nests with 35 cells were dissected, and bee food was collected from the cells (n = 35). The length and width of the bee food were measured, and the wet weight was determined. Each sample of bee food was evenly divided into three portions, and a 0.2 g specimen was weighed from each portion and dissolved in 5 ml acetone. This solution was shaken for even mixing and dropped onto a glass slide with a pipette. The morphology of pollen was observed using an Olympus STM6 optical microscope (the proportions of different types of pollen were determined under an ×10 visual field, and the plants were observed under an ×40 visual field). The observed morphologies were compared with the morphologies of the local plant pollen from the reference glass slides to determine the proportions of different pollen types in the bee food [33, 34]. Three subsamples were taken from each sample of bee food, and three visual fields were observed for each subsample under the microscope.

### Statistical analysis

Statistical analysis was conducted using Excel and SPSS 20.0. An independent *t*-test was used to compare the morphological indices of *X*. *valga* females and males and the thickness of the inner cell partitions and the outer cell partitions in different years. A paired-samples *t*-test was used to compare the thickness of the partition of the first cell with that of the outermost cell in the same nest. Pearson analyses were performed to determine the correlation between the length of the branched tunnels and the number of cells in the tunnels. *p<0*.*05* was considered to indicate a statistically significant difference. Origin 2018 was used for plotting.

## Results

### Morphology of *X*. *valga*

There were significant differences between females and males in terms of wingspan (*df*_(1,15)_ = 5.157, *p*<0.01) and body length (*df*_(1,15)_ = 3.973, *p* = 0.001). There was no significant difference between females and males in terms of head width (*df*_(1,15)_ = 1.696, *p* = 0.111), but there was a significant difference between females and males for head length (*df*_(1,15)_ = 2.467, *p* = 0.026). There was no significant difference between females and males for mesothorax width (*df*_(1,15)_ = 0.860, *p* = 0.403), but there was a significant difference between females and males for proboscis length (*df*_(1,15)_ = 3.201, *p* = 0.006) (Table 1; S1 File).

**Table 1:** Morphological characteristics of *X*. *valga* (mm).

Index	Female (n = 8)		Male (n = 9)	
	Range	Mean+SE	Range	Mean+SE
Wingspan	41.03–46.26	43.96±0.55	37.53–42.29	39.83±0.57*
Body length	20.04–24.7	23.07±0.55	17.72–22.18	20.20±0.47*
Head width	5.59–6.96	6.42±0.19	4.55–7.54	5.73±0.33
Head length	4.89–6.51	5.65±0.63	4.06–5.99	4.81±0.25 *
Mesothorax width	6.19–8.91	7.51±0.30	6.07–8.45	7.16±0.27
Proboscis length	6.87–8.03	7.59±0.16	6.34–7.75	6.89±0.15*

* denotes a significant difference compared with females for the same index.

### Nesting location and substrate of *X*. *valga*

**Fig 2:**
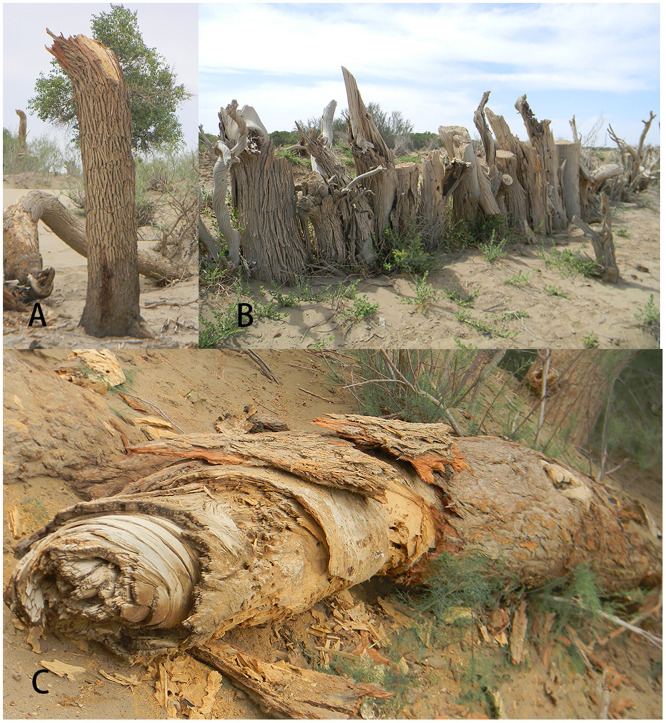
Nesting location and substrate of *X*. *valga* A. Standing dead wood; B. Dead stump; C. Dead wood lying on the ground.

**Fig 3:**
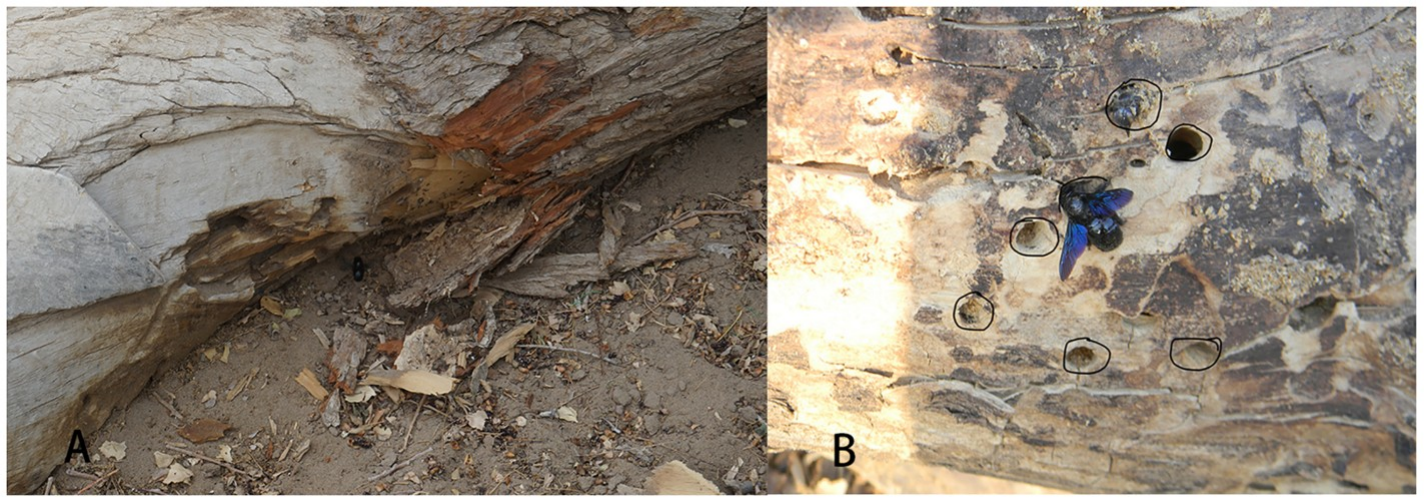
Nests in dead wood lying on the ground were located on the shaded side (A); the distance between nests (B).

*X*. *valga* builds its nests in the dead wood of *P*. *euphratica* in the *P*. *euphratica* forest reserve in Ejina Oasis, Inner Mongolia. The locations of the nests were variable. Nests of *X*. *valga* were found in standing dead trees, standing stumps and dead wood and branches lying on the ground (Fig 2). All nest entrances in standing dead trees and standing stumps had a southern exposure, and those in dead wood lying on the ground were located on the shaded side, where the direction was unknown (Fig 3A). The nests of *X*. *valga* exhibited an aggregated distribution in the wood of *P*. *euphratica*. In each piece of dead *P*. *euphratica* wood, dozens of nest entrances were generally observed (because the number of aggregated nests was large and the discarded old nests and new nests were interspersed, exact numbers were difficult to obtain). The distances between nests were short, with the closest pair located within 2 cm of each other (Fig 3B).

### Development of *X*. *valga* in the nest

Between May 22 and May 31, 2019, females were observed to select locations for the building of nests at their nesting sites. Between June 1 and June 10, females collected pollen for fermentation to produce food and laid eggs. Among the 35 dissected cells that were observed, 65.71% contained eggs, 25.71% contained immature larvae, and 8.58% contained dead eggs or no eggs. Among the 24 cells dissected between July 1 and July 10, 2014, 95.83% contained larvae (39.13% of the larvae were parasitized), and 4.17% contained pupae. Among the 11 cells dissected from July 22 to July 31, 2015, 36.36% contained white pupae, 45.45% contained black pupae, and 18.18% contained newly emerged adults. Between August 22 and August 31, 2015, newly emerged adults were observed visiting flowers in the wild. Based on the developmental progress of the offspring as well as the outside activities of the descendant emerged adults, *X*. *valga* is univoltine, and offspring overwinter as adults.

### Nest architecture and structure of *X*. *valga*

*X*. *valga* burrows into the dead xylem layer of *P*. *euphratica* to excavate a nest entrance and then digs a tunnel inward to build a nest. The nest tunnel dug out by the first *X*. *valga* female in dead wood is the closest to the outer part of the xylem layer. With an increase in nest density, females gradually build their nests toward the interior of the xylem layer. It is difficult to determine the nesting history of a family in a dead wood segment with a high nest density.

**Fig 4:**
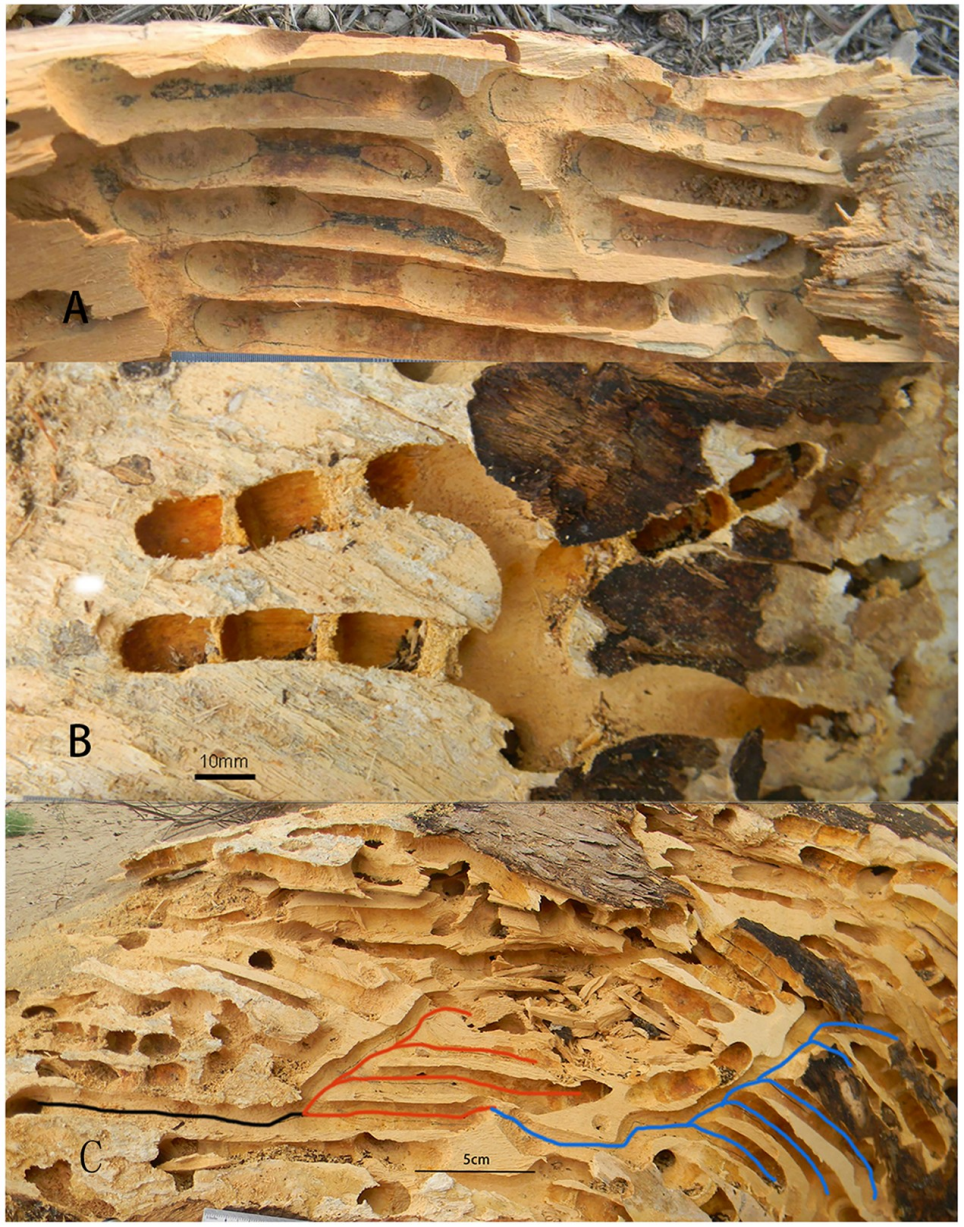
Nest structure of *X*. *valga*. A. Parallel branched type; B. Forked branched type; C. Newly emerged females use one tunnel branch of the maternal nest to continue to dig the nest tunnel. The black line indicates one branch of the maternal nest. Red lines indicate a nest built off a branch of the maternal nest. Blue lines indicate a second nest built off the red nest.

The nest entrances of *X*. *valga* were nearly circular, with an average vertical diameter of 11.42 ± 0.37 mm and an average horizontal diameter of 11.67 ± 0.36 mm (Table 2). The nests belonged to the branched type, consisting of parallel branched nests (82.61% of the 23 nests dissected in 2014 and 2015) (Fig 4) and forked branched nests (17.93%). The number of branches in the *X*. *valga* nests ranged from 1–5, with 3 branches being the most common, accounting for 45.83% of the surveyed samples (n = 24), followed by 1 branch (8.33%) and 5 branches (4.17%). The average length of the branch tunnels in a nest was 68.43 ± 4.37 mm (Table 2).

**Table 2:** Parameters of the structure and composition of nests of *X*. *valga* in the Ejina Oasis. These parameters were determined for all nests examined.

Parameter	*n*	Minimum-maximum	Mean±SE
Nest entrance length, mm	19	8.88–13.94	11.42±0.37
Nest entrance width, mm	19	8.24–13.43	11.67±0.36
Cell length, mm	42	13.65–20.8	17.29±0.25
Cell diameter, mm	42	8.97–14.11	11.24±0.20
Cell number per nest	23	4–10	7.35±0.35
Branch number per nest	24	1–5	3.08±0.19
Cell number per branched tunnel	58	1–5	2.52±0.12
Branched tunnel length, mm	37	36.33–144.22	68.43±4.37
Cell partition (inner), mm	38	1.28–3.33	2.32±0.09
Cell partition (outermost), mm	20	3.68–12.35	6.61±0.52

The parameters were calculated for the 43 nests dissected in 2014, 2015 and 2019. The number (n) in this table refers to the samples that could be measured or counted, excluding samples that could not be assessed due to damage during nest dissection or an overly complex nest structure.

The length of the tunnel excavated by the female generally ran parallel to the texture of the wood material. The newly emerged females used one branch tunnel of the maternal nest to continue to dig the nest tunnel inward (or forward) (Fig 4C). Several females used the same nest entrance. There was no communication between females, as they built their own cells separately. Alternatively, *X*. *valga* females could choose a new location to build a new nest (Figs 2 and 4). Among the 43 dissected nests, 5 were built by reusing the maternal nest, with a utilization rate of 11.63%.

The cell number per branched tunnel ranged from 1–5, with a mean of 2.52 ± 0.12. The most common cell number per branched tunnel was 2, accounting for 41.38% of cases, followed by 3 cells, accounting for 37.93% (Table 2). Pearson correlation analysis revealed a significant correlation between the length of the branched tunnel and the cell number per branched tunnel (r = 0.660, *p* = 0.001). The total number of cells per nest ranged from 4–10, with a mean of 7.35±0.35.

**Fig 5:**
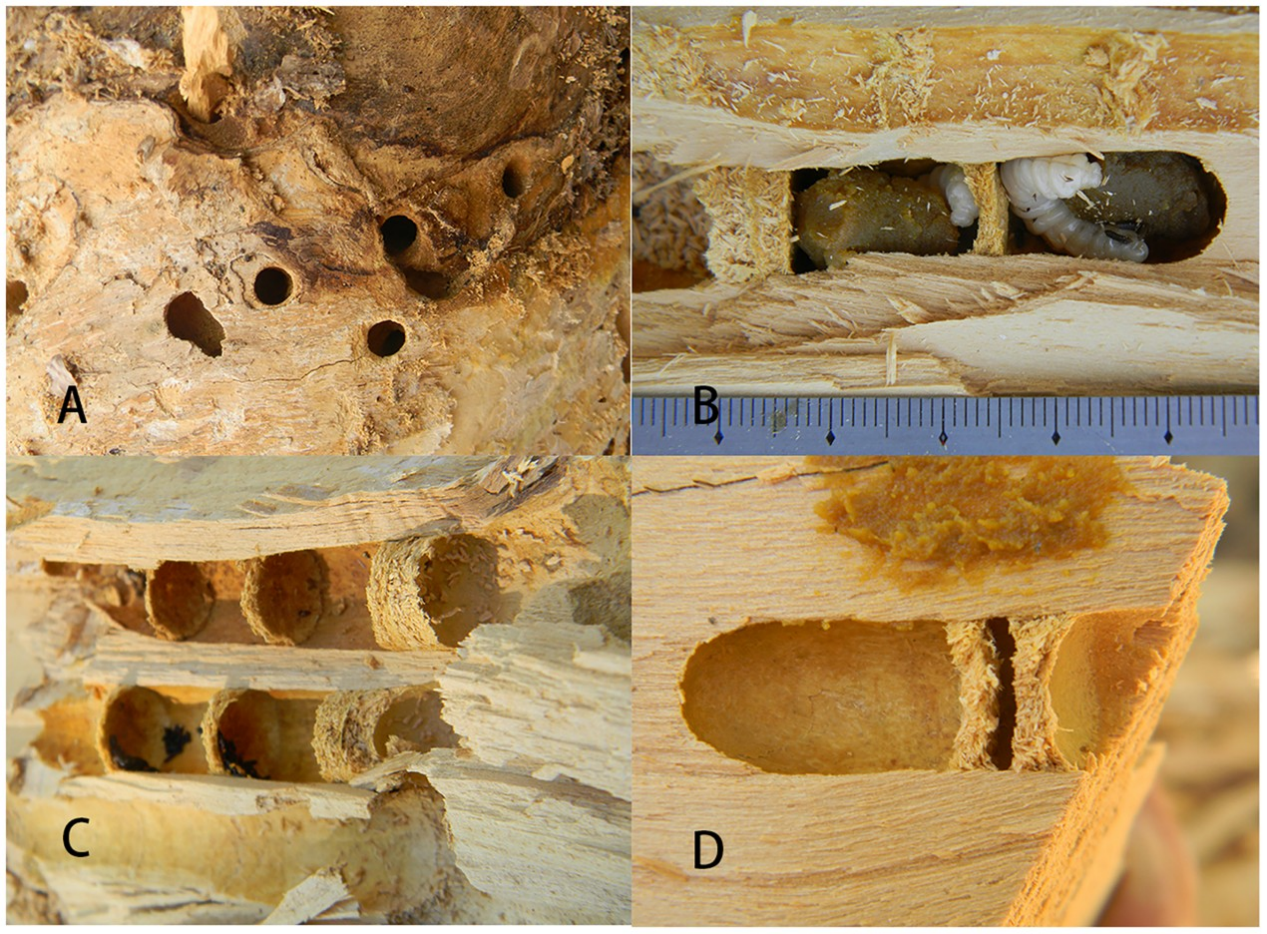
Nest architecture of *X*. *valga*. A. Nest entrance; B. Nest structure of the branch tunnel; C. Structure of the inner cell partition and outer cell partition within the branch tunnel; D. Dual partitions of the outermost cell.

After the excavation of a branch tunnel was completed, *X*. *valga* collected pollen and nectar to produce bee food, and the females laid eggs on the bee food. Next, wood chips were excavated near the nest entrance to form a cell partition in order to block off the cell. The cells of *X*. *valga* exhibited a long cylindrical shape. The average length of the cells was 17.29±0.25 mm, and their average diameter was 11.24 ± 0.20 mm (Table 2). A cell partition separated one cell from another. The inner side of the cell partition was coarse, and the outer side was smooth. The thickness of the inner cell partition of the branched tunnel was 2.32±0.09 mm on average, while that of the outermost cell partition was 6.61±0.52 mm (Table 2; S2 File). There was a significant difference between the thickness of the first cell partition of the branch tunnel and that of the outermost cell partition (paired-samples *t*-test, *df*_(1,19)_ = -9.125, *p*<0.001) (Table 3, and Fig 5). There was a significant difference between the thickness of the outermost cell partition of the branch tunnel recorded in 2014 and that recorded in 2019 (independent-samples *t*-test, *df*_(1,19)_ = -2.969, *p*<0.001), whereas no significant difference in the thickness of the first cell partition of the branch tunnel was observed between years (independent-samples *t*-test, *df*_(1,18)_ = -2.753, *p* = 0.230) (Table 3). There was a significant difference between the thickness of the first cell partition and that of the outermost cell partition in both 2014 and 2019 (independent-samples *t*-test, *df*_(1,18)_ = -2.352, *p* = 0.030) (Table 3). Double partitions of the outermost cell were also found in June 2019 (Fig 5D).

**Table 3:** Thickness of *X*. *valga* cell partitions in different years (mm).

Year	Branch no.	First cell partition	Last partition	Thickness difference
July 2014	01	1.56	5.39	3.83
	02	1.83	5.83	4
	03	2.27	5.57	3.3
	04	1.71	5.4	3.69
	05	1.74	6.15	4.41
	06	1.65	6.45	4.8
	07	3.03	6.17	3.14
	08	3.05	5.96	2.91
	09	1.85	5.89	4.04
	10	1.79	4.46	2.67
	11	2.67	5.7	3.03
	12	1.76	3.68	1.92
	Average	2.08±0.16	5.55±0.22*	3.48±0.23
June 2019	01	2.51	4.65	2.14
	02	2.97	10.77	7.8
	03	2.44	4.44	2
	04	2.95	9.88	6.93
	05	3.33	8.94	5.61
	06	2.78	8.98	6.2
	07	2.28	12.35	10.07
	08	2.24	5.54	3.3
	Average	2.69±0.14	8.19±1.05*	5.51±1.01

* denotes a significant difference in thickness between the first cell partition and the outermost partition in the same observation year.

### Bee food storage

*X*. *valga* females collected pollen and nectar, which they fermented to form bee food. In this study, we collected 35 pollen balls in total. The wet weight of the bee food ranged from 0.9926 g to 1.9950 g, with a mean of 1.1210±0.0385 g (n = 35) (S2 File). Robust measurements and observations were completed for only 12 balls due to damage during sample treatment. The bee food was nearly rectangular in shape. Its length ranged from 15.28–18.77 mm, with a mean of 16.71±0.31 mm, and its width ranged from 9.93–12.19 mm, with a mean of 11.05± 0.22 mm.

**Fig 6:**
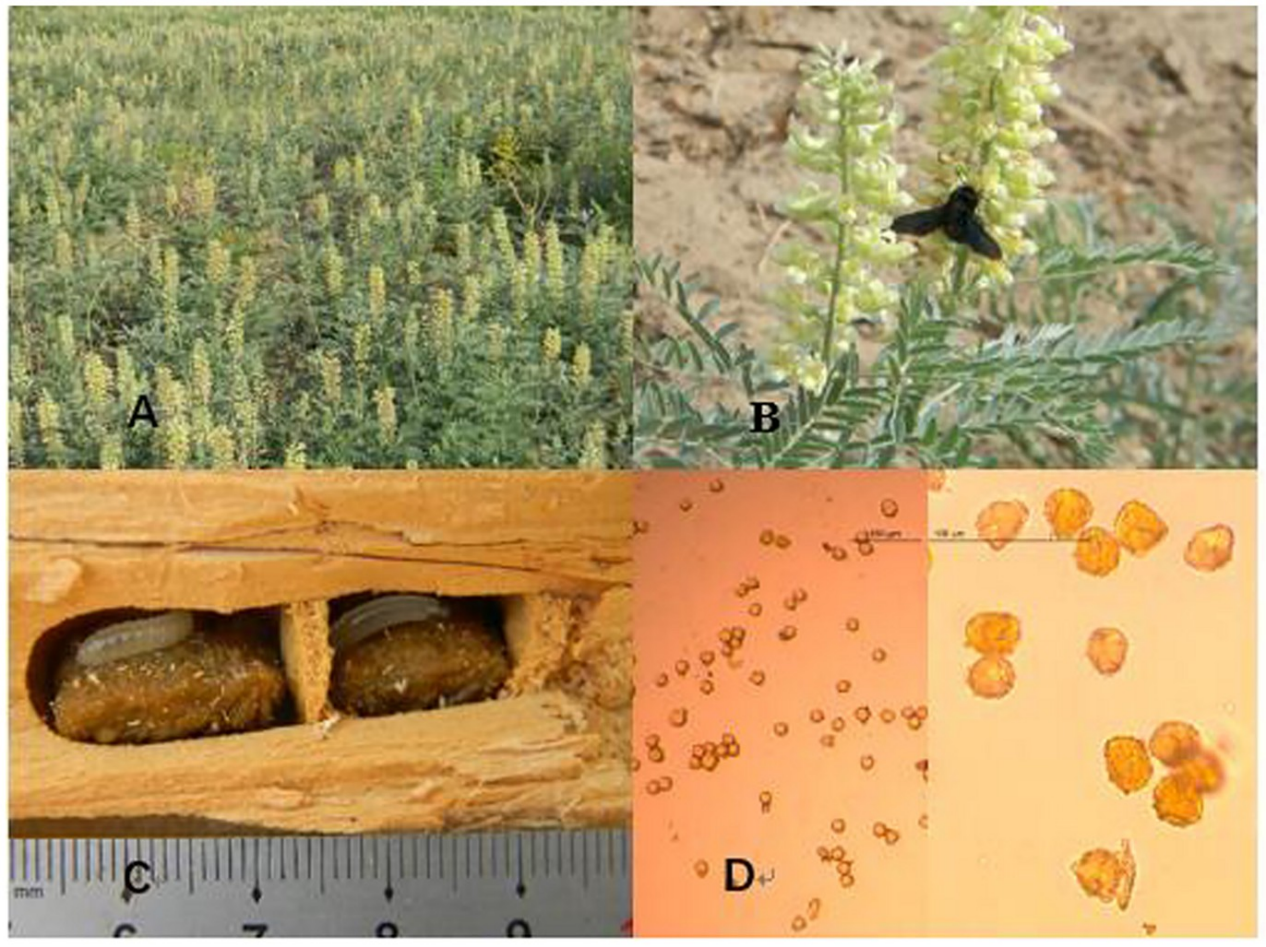
Pollen fermented by *X*. *valga* to produce bee food under an Olympus STM6 optical microscope (×40). A. Population of *S*. *alopecuroides* in full bloom; B. *X*. *valga* females visit *S*. *alopecuroides*; C. Bee food stored by *X*. *valga* in the dead wood of *P*. *euphratica*; D. Pollen of *S*. *alopecuroides* in bee food.

According to optical microscopy, the pollen of *S*. *alopecuroides* accounted for 84.63±1.21% of the total bee food, that of *S*. *salsula* accounted for 6.79±0.65%, and unidentified pollen types accounted for 8.58% (Fig 6).

## Discussion

In this study, we investigated the nesting behavior of *X*. *valga* and its pollen collection for bee food fermentation in the *P*. *euphratica* forest reserve in the Ejina Oasis, China, and found for the first time that *X*. *valga* nests in dead *P*. *euphratica* in the wild. Additionally, we found that the population of *X*. *valga* is locally dense and shows the notable characteristic of nest aggregation. These characteristics of nesting are presumably associated with the softness and strong antirotting properties of the wood fibers of *P*. *euphratica*. According to a literature search, no reports on the nesting behavior of *X*. *valga* in China have been published. Nevertheless, our team has also investigated the nesting behavior of these bees in some regions of Xinjiang and Gansu. In Manas and Altay of Xinjiang and Gaolan County of Gansu, the nests of *X*. *valga* have mainly been found in wooden beams and livestock sheds, and its nest substrates include poplar, catalpa and willow wood. In addition, in Manas (86°12.6044E, 43°50.3961N; altitude, 1573 m) and Altay (86°25.4975E, 48°09.5713N; altitude, 592 m), we found that *X*. *valga* nested in soil. These observations, combined with those in the literature [8, 35], suggest that *X*. *valga* shows high adaptability and flexibility in the selection of nesting locations. However, the differences in its nesting behavior in dead *P*. *euphratica* versus wooden buildings and structures and soil as well as the influence of these differences on *X*. *valga* population structure remain to be explored.

*Xylocopa* species may nest in dead wood because they exhibit a strong preference for this type of nest substrate [15] or because of limited nest substrate resources [17]. Within the nest substrate, Xylocopa bees often form a high-density agglomeration of dozens or even hundreds of nests with a very short distance between nests [17]. Offspring normally expand the maternal nest to form multiple independent nests, and multiple female bees use the same nest entrance [15]. However, this high-density, aggregated lifestyle increases nest site competition and pressure related to nest entrance recognition, intranest behavior and natural enemy defense [17]. Under the conditions of high-density aggregation, *X*. *varipuncta* can rapidly recognize the nest entrance after foraging based on visual and olfactory cues [17]. How *X*. *valga* rapidly recognizes the nest entrance, avoids competition with individuals of the same species and avoids natural enemies needs to be investigated.

Bee cells are shelters that protect vulnerable offspring as well as food for the development of larval offspring [1]. *X*. *valga* constructs partitions to block its cells, which is similar to the behavior of other *Xylocopa* species [11, 22, 15, 27, 36]. However, unlike the pattern observed in other *Xylocopa* species [21, 25], the partition of the outermost cell constructed by *X*. *valga* was significantly thicker than that of the inner cells. Furthermore, the outer partition of each main tunnel was significantly thicker than the innermost partition. Although the thickness of the first cell partition in the nests of *X*. *valga* in 2014 was comparable to that in 2019, the outermost cell in 2019 was significantly thicker than that in 2014. In addition, we found double partitions of the outermost cell in 2019. The underlying nesting mechanism that explains why *X*. *valga* significantly increases the partition of the outer cell remains unclear and should be explored in the future.

Pollen is the main ingredient in the food stored by the mother bee, which serves as the sole nutrition source for bee larvae [19, 37]. Different carpenter bee species collect different types of pollen. There are also differences in the kind of pollen collected by the same kind of carpenter bee in different distribution areas. In Argentina, 18 kinds of pollen have been reported in *X*. *augusti* food [38]. Twenty-nine pollen types were identified in the food of *X*. *nasalis*, 13 of which were the main pollen sources [39]. *X*. *valga* uses a variety of plants as food sources [8]. This study was conducted in the Ejina Oasis of China, where the type of pollen found in larval food of *X*. *valga* was relatively homogenous. According to our results, the pollen of *S*. *alopecuroides* accounted for over 84% of the total bee food samples, which might be related to the species and distribution of nectariferous plants in the *P*. *euphratica* forest area. The influence of *S*. *alopecuroides* on the health of *X*. *valga* and the development of the offspring may be another interesting research direction in the future.

The *P*. *euphratica* forest in the Ejina Oasis is one of the main natural *P*. *euphratica* forests in the typical desert area of China and is the only forested area in western Inner Mongolia. The forest also constitutes an ecological barrier that helps maintain the Ejina Oasis and has considerable ecological and scientific significance [28]. *S*. *alopecuroides*, which is characterized by strong drought and alkali resistance and fast growth, is a pioneer plant used as a windbreak and for sand fixation in the *P*. *euphratica* forest area [30]. Its flowering period lasts from May to June, and its full-blossom period is from June 1 to June 20. Our study found *X*. *valga* nests only in dead *P*. *euphratica* and that *S*. *alopecuroides* in full bloom is the major food resource used by *X*. *valga* to nourish its progeny. Our results reveal that *P*. *euphratica*, *S*. *alopecuroides* and *X*. *valga* show a close ecological relationship, laying a foundation for further investigations of the interaction between species in the *P*. *euphratica* ecosystem.

## Conclusions

This study is the first to report that *X*. *valga* nests in dead *P*. *euphratica* in the Ejina Oasis, China. *X*. *valga* is univoltine and lives alone in cells, with multiple females using the same nest entrance. The bees produce branching-type cells, in which the partition of the outermost cell is significantly thicker than that of the inner cells. In Ejina, the dominant nest substrate for *X*. *valga* is dead *P*. *euphratica*, and the dominant food source is *S*. *alopecuroides*. Additionally, *X*. *valga* is the dominant pollinator of *S*. *alopecuroides*. The results of this study may lay a foundation for future exploration of the relationships between the population structure of *X*. *valga* and its nesting and food resources. In addition, this study may provide useful data for future *X*. *valga* conservation efforts.

## Supporting information

S1 File Morphological data for the female and male bees. (XLSX)

S2 File Raw data on the thickness of the inner and outermost cell partitions and the bee food. (XLSX)
